# Supplementary material for: Self reward design with fine-grained interpretability
Source: Sci Rep. 2023 Jan 30;13:1638. doi: 10.1038/s41598-023-28804-9 (PMC9886969; doi:10.1038/s41598-023-28804-9)
Supplement: Supplementary file 1 — Supplementary Information. [file 41598_2023_28804_MOESM1_ESM.pdf]

Self Reward Design with Fine-grained Interpretability

Erico Tjoa, Cuntai Guan

Appendix

This Paper Focuses Heavily on Interpretable Human Design

We reorganize this paper heavily based on ICLR 2022 reviewers' comments and have further revised the majority of this paper based on Scientific Report reviewers' comments. Thanks to them, we understand that our focus on fine-grained interpretability (through direct manipulation of weight and biases) seems to have been lost or easily overlooked in the previous version of our paper. There is a tendency to focus on performance and solution to generalizability. We have answers for this (1) we do achieve high performance, and comparison might be very redundant. Our intention is to compare interpretability instead of performance. (2) Scalability. We naturally start by demonstrating the design on simple problems. Starting with a highly complex problems may be counterproductive and very difficult to present. In this revised paper, we have focused on the clarity of presentation.

Convergence. The design process should ideally require step-by-step testing that ensures the "convergence" of the model, which we demonstrate through our solution to MuJoCo half-cheetah. The PFC module that facilitates optimization should also be tested to ensure that the conceptual designs are working. Also, stable convergence is no longer guaranteed[?] in most cases when non-linear functions are involved.

Why do the weights and biases remain interpretable after SRD optimization? In our SRD framework, the initial selection of weights and biases are required to work to an extent, which means that the loss w.r.t a given metric of the problem is not very high from the very start, or at least the loss is not as high as randomly initialized model. This perhaps means that the designed weights and biases are already somewhat near a local minimum point. In practice, the SRD optimization probably only finetunes the parameters so that the model moves slightly nearer the local minimum, thus not changing the weights and biases too much."

Related Works

In Reward Design Problem[26], it is observed that, given bounded agents, proxy reward function can be more optimal than the true fitness function which is distinct from the proxy. Dylan's IRD, on the other hand, approximates the true fitness function given an observed proxy reward function, assuming the designer is the bounded agent, i.e. the designer is fallible. IRD performs inversion to compute $P(w=w^*|\tilde{w},\tilde{M})$ from $P(\tilde{w}|w^*,\tilde{M})$, where w is the weight of the reward function $r(\xi;w)$, where ξ is the trajectory. We cannot fit the description of our model exactly in the same language, since we opt for an interpretable design with non-traditional RL reward. The closest we have to a reward is v_{plan} in Robot2NN, shown in fig. 5 indirectly as the values summed through orange arrows in v_Σ described in the next paragraph.

Comparison with standard Deep RL. In the main text, we mention that our we use non-traditional reward function. Deep Q-learning loss function $L_i(\theta_i)$ computes differences between current Q value (value of the current state-action pair) and its temporal difference (TD)[1] given by $r+\gamma max_{a'}Q(s',a',\theta_{i-1})$. The reward r can be fixed by the designer; this can be seen on OpenAI's official github[2], and the values are then used to update Q, an expected value of total future rewards, technically also a reward. SRD is similar to DQN in this sense: there is a future cumulative reward. In turn, DQN is similar to standard RL except Q is predicted by the neural network. However, SRD does not apply the loss function $L_i(\theta_i)$ in DQN since we do not specify TD, which is dictated by the reward that explicitly adds into the sum of future reward. As mentioned, the reward value r in DQN is specified by the designer and thus DQN robot aims to maximize values whose absolute magnitude of worth is known. By contrast, SRD lets the robot measure the worth of every series of actions. Since the target Tanh score is 1, every measurement of worth is relative with respect to the learning process. Even then, when the robot compares the multiple plans it makes at once, it has its own yardstick for rewarding its own decisions. The absolute magnitude of reward based on this yardstick is decided by the robot's own neural network, thus the "self reward". Finally, our robot self reward works by always undervaluing its perceived reward w.r.t the ideal plan with score 1, as mentioned in the main text.

Self-supervision. Self-supervised DRL is demonstrated on a simulated car that learns from real-time experience[21]; the paper also applies the model to a real-world RC car. After hybrid graph-based RL model is incorporated, it travels around avoiding collisions without human intervention. Our robots self-supervise similarly. After the initial interpretable design, 2D Robot moves towards the target, favoring or avoiding different types of tiles without human supervision. In particular, grass tiles are recognizable, but less desirable, while lava tiles are "not recognized", hence must be completely avoided. Informally, the robots have their own preferences on how to assign the eventual values of the actions with known local consequence, while they are careful about their own ignorance induced by imperfect design (unknown avoidance).

[1] see for example https://www.tensorflow.org/agents/tutorials/0_intro_rl

[2] https://github.com/openai/gym/blob/master/gym/envs/classic_control/cartpole.py

Imagination components. Unlike an existing rollout[27], each of our SRD rollout consists of a series of asymmetric binary choices a_1, a_2 chosen from $\{a \in \mathscr{A}\}$ so that $v(a_1) \geq v(a_2)$, where $\mathscr{A}$ is the set of actions and v is any generic function that gives each action a local value. The values will be aggregated into a self-reward. Dreamer[28] solves RL problem using only latent imagination where many models (such as reward, action and value models) are specified as probability distributions. By contrast, SRD creates no specific sub-model. All values are just NN activations, and they will be aggregated into impromptu, just-in-time scores, based on which the plans can be greedily chosen.

2D Robot in Lavaland

In the main text, we briefly discussed 2D robot in the lavaland. We will elaborate further here, starting with the interpretable components used in 2D robot's design:

1. The ABA: approximate binary array. With *selective activation* and tile-specific values (colour), we create strong neuron activations that specifically correspond to tile colours. Their visual maps correspond directly to the relevant signal, preserving the ease of readability.
2. The DeconvSeq. The series of convolutional kernels are intended to provide targeted response in conjunction with ABA e.g. in fig. 5, $[v_1]_{target}$ gives strong signal centred around the target. This is done by manually setting the center value of the weights to be higher than the rest (see fig. 7). The main selling point is their tunability. The weights are trainable: while each module has been given a specific purpose, e.g. detect target, it is still tunable. We empirically show that the main purpose the kernels' weights are preserved (i.e. center value still highest) after optimization.

The interpretable design of Robot2NN is shown in the main text fig. 5. Tile-based modules in the receptors are designed to respond to different types of stimuli (grass, ground, lava etc); as our previous examples, weights and biases are manually selected. Deconvolutional layers are used in the robot's PFC to give the tiles some scores for the robot to decide its subsequent action (red better, blue worse). More precisely, a stack of deconvolutional layers DS_t^n (defined later) will be used to create a *favourability gradient*. Robot then chooses an action that generally moves it from blue to red regions.

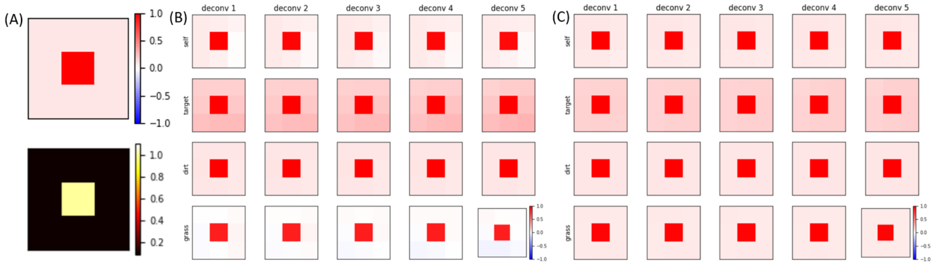

Figure 7. (A) Initial parameters; all deconvs in all DeconvSeq are initialized to the same 3x3 weights with center max value of 1 and 0.1 elsewhere. (B) Once trained, variations of weights are observed for Project A expt 1 (all 180 parameters are shown). (C) Similar to (B) but for project Compare A.

Before we proceed, we clarify some of our notational uses. ABA: approximate binary array, an array whose entries are expected to be ≈ 0 or 1. DS_t^n, or DeconvSeq, is the sequence of n deconvolutional layers for a tile type t. Deconvolutional layer, or deconv, is a regular DNN module. Each deconv is followed by Tanh activation for normalization and non-linearity. Normalization (to magnitude 1) ensures that DeconvSeq compares action choices in relative terms. Tile t denotes the name of a tile, e.g. grass, but it also denotes its $[0,1]$ normalized RGB value, e.g. for grass, $t = [0,128,0]/255$ or an array of tile values (they should be obvious from the context). $\tau_{recog} = 10^{-4}$ is the recognition threshold. Designer needs to specify $P = \{p_t : t = target, grass, ...\}$. For now P is the set of untrainable parameters, each p_t roughly acting as the factor for scaling the true reward. They will affect the robot's final preferences for or against different tiles although tunable parameters will accentuate or attenuate them accordingly. They induce the biases that designers input into the model in a simple, interpretable way. We also define the *unknown avoidance* parameter $u_a \geq 0$.

Interpretable tile-based modules are designed to explicitly map robot's response to each specific tile type. Get_ABA() function computes the ABA for each tile type: $w_t = \sigma_{sa} \circ \mu[(x_{attn} - t)^2]$ where $\mu[.]$ is the mean across RGB channel. This is reminiscent of eq. 1; the difference is, each neuron responds to a tile type at each x_{attn} coordinates. Neuron activations are computed as w_η where η =target, grass or dirt shown in fig. 5, e.g. strong activation for grass detection occurs at $[w_{grass}]_{14}$.

Unknown avoidance. Like Dylan's IRD, we have a reliable mechanism for unknown avoidance, the Boolean array $w_{unknown} = [(1 - \Sigma_t w_t) > \tau_{recog}]$ to be treated as floating point numbers. From the formula, it can be seen that $w_{unknown}$ aggregates the negation of known activations. The unknown in our case is the lava tile that the imperfect human designer 'forgets' to account for.

Interpretable tile scores. The score v_Σ will be used to decide what actions robot will take. It is computed as the following. First, we compute v_1, whose components are $[v_1]_t$, as the following. For target tiles and "self" tiles (original positions), $[v_1]_t = p_t DS_t^5[w_t]$. For any other named tiles, we also create the *favourability gradients* based on the initial and target positions, so that, for example, a grass tile nearer the target can be valued more than a dirt tile. Thus,

$$[v_1]_t = p_t DS_t^5[(w_{target} - w_{self}) * w_t] \quad (3)$$

Each deconv in DeconvSeq consists of 1 input and 1 output channel. It is fully interpretable through our manual selection of kernel weights: kernel has size 3 with center value 1 and side value 0.1 as seen in fig. 7(A). Fig. 7(B, C) show examples of trained weights. This choice of values is intended roughly to create a centred area of effect, where the center of the tile contributes most significantly to w_t; see for example $[v_1]_{target}$ in fig. 5. Assign $v_\Sigma \leftarrow \Sigma_t [v_1]_t$; this value will be dynamically changed throughout each plan-making process.

Making one plan. Each plan is a series of actions, and each action is chosen as the following. Set the specific values for the target tile and tile of the original position to v_0 and $-v_0$ respectively, where $v_0 = max\{|v_\Sigma|\}$ is computed without backpropagation gradient to prevent any complications. Finally, to incorporate lava avoidance, or generally avoidance of anything previously unseen, $v_\Sigma \leftarrow v_\Sigma * (1 - w_{unknown}) + -u_a * v_0 * w_{unknown}$. From the current position, robot's neighbouring $[v_\Sigma]_i$ where $i = up, down, left, right$ values are collected, and neighbours with the top two values are chosen. From the top two choices, randomly choose one of them with a 9 to 1 odds, favouring the action with higher value. The randomness is to encourage exploration. After each action, the tile the robot leaves will be assigned $0.9v_0$ to prevent the robot from oscillating back and forth, where v_0 is separately computed inside step_update() function. A series of actions are chosen in this manner until the target is reached or a maximum of 36 iterations are reached. Thus we have obtained a *plan* and its score $v_{plan} = \frac{1}{N_\xi} \Sigma_{i,j \in \xi} [v_\Sigma]_{i,j}$, the mean of all values assigned to the chosen tiles where ξ is the trajectory (see fig. 5, orange arrows in v_Σ).

Imagining multiple plans for SRD optimization. Robot makes plans by imagining $n_{plans} = 4$ different trajectories to reach the target. It plans and executes the plan with the highest v_{plan}. Like[19], we use all n_{plans} imagination branches for training (SRD optimization) with possibly novel loss minimization:

$$loss = \Sigma_{i=1}^{n_{plans}} (1 - Tanh(N[v_{plan,i}]))^2 \quad (4)$$

where $N[.]$ normalizes v_{plan} to the magnitude of 1, where normalization factor is computed without gradient to prevent complication. Thus, robot's PFC always undervalues its reward relative to an abstract, ideal value 1. The intended effect is to always try to maximize v_{plan}. A standard Stochastic Gradient Descent with learning rate 10^{-4} is used for optimization. This resembles fish's PFC true or false neurons used for rewarding itself, except it is continuous.

Experimental setup and comparison. We experiment on several initial settings as the following. For each experiment, we randomly generate and save 4096 maps to evaluate the performance of both standard design and SRD trained model, and similarly 4096 maps for SRD training. Note: All codes are included, including codes for creating animated gifs of robot traversing the lavaland.

Reaching targets without training, optimized by training. Table 2 shows that even without training ("No SRD" columns), our interpretable designs have enabled relatively high rate of problem solving. With SRD, the accuracies are further improved.

Table 2. Accuracy comparisons for Project A, project Compare A, project With-Lava-A and project Lava NOAV A. No. 1 to 4 indicate four independent but identical experiments. $Acc = n_{reached\ target}/4096$. "No SRD" indicates no training: reasonable accuracy is attainable purely with design. SRD training generally increases the accuracies. Starred values reflect failure modes, matching their respective histograms; see fig. 8 and 9.

	Project A		Compare A		With Lava A		Lava NOAV A	
	No SRD	SRD	No SRD	SRD	No SRD	SRD	No SRD	SRD
1	0.767	0.856	0.862	0.941	0.826	0.893	0.841	0.884
2	0.765	0.845	0.871	0.589*	0.821	0.893	0.850	0.909
3	0.763	0.817	0.874	0.930	0.819	0.837	0.844	0.868
4	0.760	0.868	0.863	0.422*	0.823	0.762*	0.835	0.907

Comparison of results on different settings:

1. **Project A: standard design and SRD on maps without lava tiles**. The fraction of grass tiles is 0.3. No robot will be spawned at the target immediately. Here, we use $p_{dirt,grass} = 0.2, -0.8$ chosen empirically. For training, we only run through

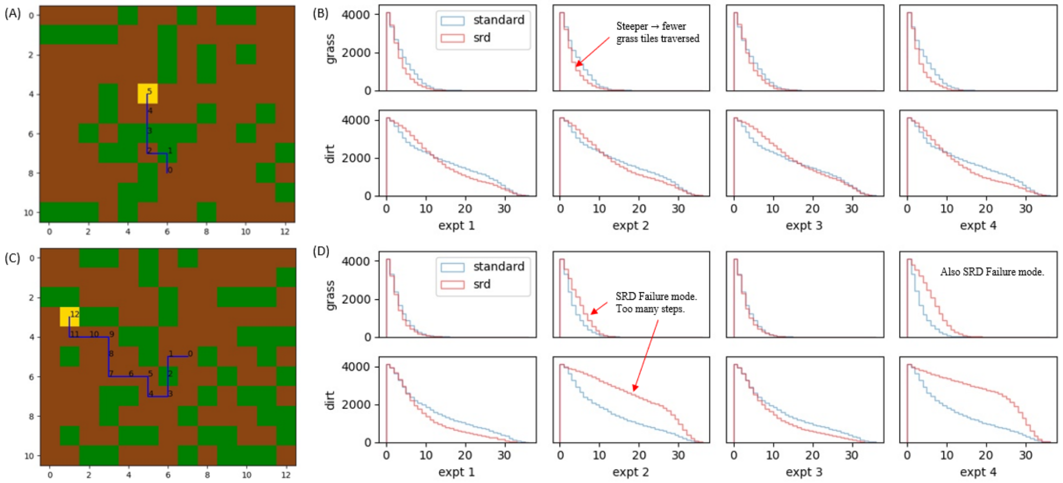

Figure 8. (A) A sample trajectory from Project A. (B) Cumulative histogram of the no. of tiles traversed by robot in Project A. Steeper histogram indicates that less tiles of the type are being traversed (for grass, steeper is better) (C) A sample trajectory from Compare A. (D) Similar to B, but for robot in Compare A.

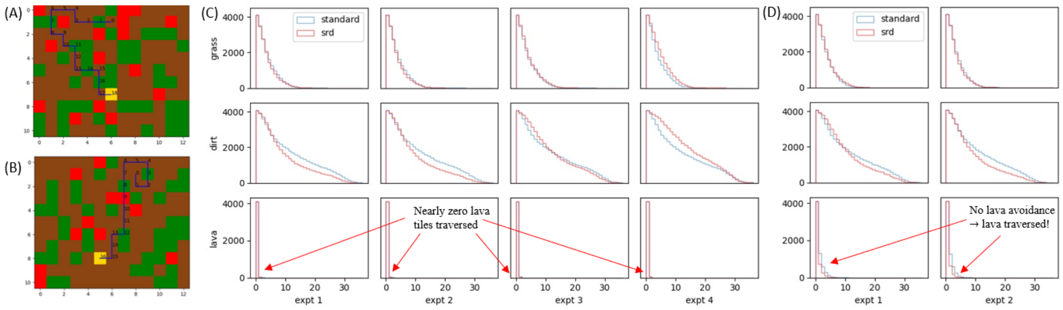

Figure 9. (A) A sample trajectory from Lava A. (B) A sample trajectory from Lava NOAV A. A lava tile is traversed. (C) Cumulative histogram of the no. of tiles traversed by robot in Lava A. Almost zero lava tiles are traversed. (D) Similar to (B) but for Lava NOAV A, in which some lava tiles are traversed.

1 epoch, taking a very short time to complete (less than 0.5 hour per project without GPU). Fig. 8(B) shows the cumulative histogram of the number of tiles traversed with $p_{target} = 2$.

2. **Compare A: model is more focused on the target**. This setting is similar to Project A, but we use a more extreme parameter setting, i.e. set $p_{target} = 10$. The local reward of the target tile is increased, and from table 2, we see that this generally increases the accuracies of both standard and SRD trained model (more likely to reach the target tile). The value seems to produce more unstable results as well, i.e. we empirically observe more failure modes.
3. **Project Lava A**. In this experiment, 0.1 of the tiles randomly assigned as lava and unknown avoidance $u_a = 2$. We also set $p_{target} = 10$ thus, similar to *Compare A* we see higher accuracy but some failure modes as well. The model is designed to not recognize a lava tile, so a lava tile will activate the robot's Robot2NN $w_{unknown}$ at lavas' positions. Fig. 9(A) is just an example of how robot successfully avoids lava, while fig. 9(C) lava cumulative histograms show that there are nearly zero lava tiles traversed.
4. **Project Lava NOAV A**. Project Lava NOAV A is similar to Project Lava A, but we set $u_a = 0$, i.e. no unknown avoidance. The results are clear, we see in 9(D) that robot will traverse the lava tiles without much regards.

Multiple trials of the above experiments have been conducted for reproducibility (project B, C, D etc). Each trial also consists of 4 experiments. Other results are in the supplementary materials (full version will be released later). The following are the names for the corresponding experiments. Repeat trials for Project A: project B, C and D. Histogram for project B can be seen at fig. 12. Repeat trials for Compare A: compare B, C and D. Repeat trials for Lava A: Lava B, C, D. Repeat trials for Lava NOAV A: Lava NOAV B, C, D.

Robot2NN weights and preserved interpretability. This model is very efficient because it consists of only 180 trainable parameters, as shown fully in fig. 7(B,C). As expected of relatively simple problems, there is no need for millions of parameters required to achieve high accuracy. High performance 90% accuracy is attained, given 10% randomness is allowed. The weights of target deconv appear to have been trained towards higher positive values (redder). The center value remains the most prominent for all, thus preserving our interpretability. Looking into individual variations, fig. 7(B) shows the weights from Robot2NN model of project A expt 1 while fig. 7(C) from project Compare A expt 1. The difference in grass weights are apparent.

We have seen from fig. 7 that the trained models still have the general interpretable shape we initiated it with. While there is no theoretical proof, there may be an intuitive reason. Due to our interpretable design, the model starts off with a reasonable ability to solve the problem. This probably means weights and biases already reside in a high dimensional parameter space somewhere around one of the local minima. This local minimum is special in the sense that it is more interpretable i.e. weights have recognizable shapes as we have initiated. As a result, a short training leads it nearer to that local minimum, hence the overall shape of the model remains similar to the initial shape and interpretable.

(2) Why no conclusion should be drawn from this observation? It seems that project A with smaller p_{target} results in relatively less preference for the grass tiles, which in turn leads to negative values (blue) for deconv for grass tiles. By comparison, project Compare A seems to have no negative values for grass tiles deconv. Unfortunately, the weights are shown only for demonstration; *there is no definite conclusion that can be drawn*. This is because other experiments similar to project A also can result in all positive deconv weights with different patterns. They still yield high accuracy, thus possible variations within even this small set of parameters can still produce similar performance. Other results are shown in the appendix. Lava A project does not yield particularly distinct patterns. We see that even failure modes can yield weights profile that look similar to non-failure modes. Further investigations may be necessary.

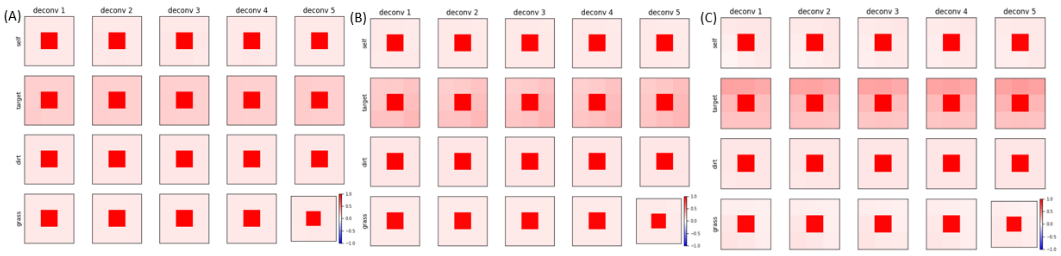

Figure 10. Weights for (A) Project Lava A expt 1 (B) Project Lava NOAV A expt 1 (C) Lava A expt 4.

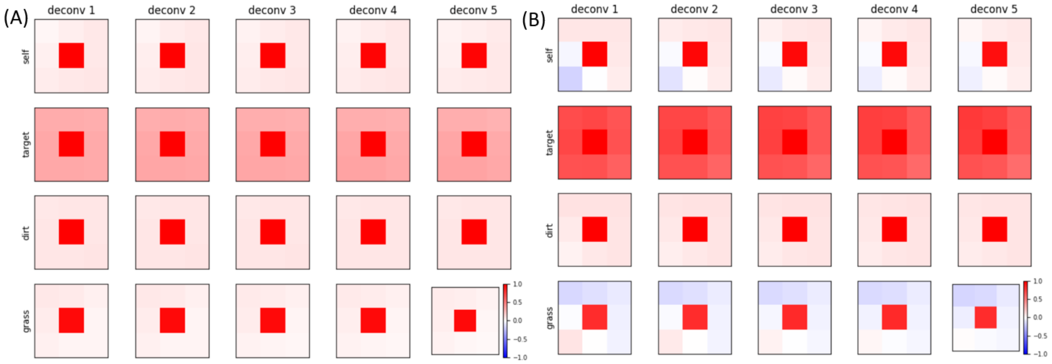

Figure 11. Weights for failure modes (A) Project Compare A expt 2 (B) Project Compare A expt 4. (A) still shows standard-looking weights.

MuJoCo with SRD

We briefly described the application of SRD framework on half cheetah simulation on MuJoCo in the main text. Here, we will go through step by step process to arrive at the HalfCheetahSRD design (executed using `--mode srd-model-design`

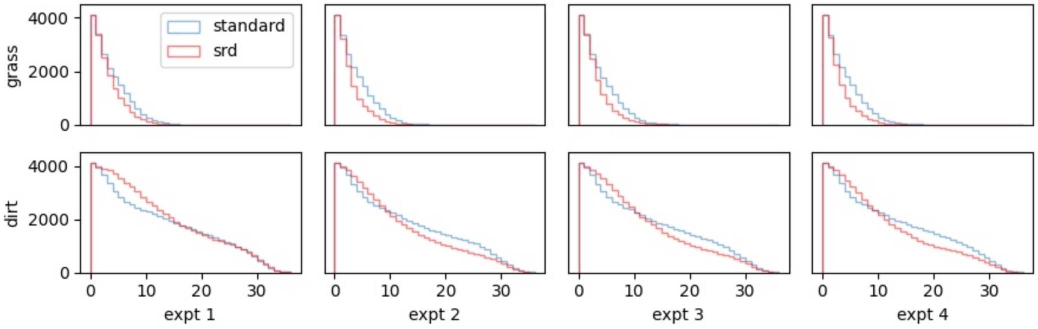

Figure 12. Project B cumulative histograms.

argument); also, see model_half_cheetah_design_stage.py. The full set of commands used to execute our experiments can be found in misc/commands_mujoco.txt.

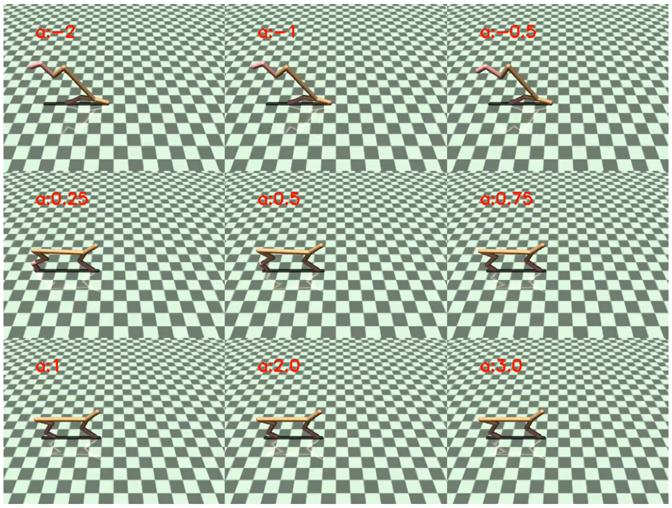

Figure 13. Half cheetah's poses after the application of varying strengths of actuators (front and back thighs).

Stage 0: devtests. To be able to design SRD properly, it is important to understand some fine details of the models and the platform used to simulate the model. We perform an initial testing to observe the agents' poses visually. Run the python command with `python mujoco_entry.py --mode devtests --testtype vary_control_strength --model half-cheetah`). Here, we arbitrarily choose a specific set of setup that we keep consistent throughout the experiment. For example, the framerate is set to 15 Hz (which does affect the time-step size) and $interval = 25$ (a setting for SRD model's momentum update). They are arbitrary; for our current experiments, all we need is for the agent to be able to stabilize and perform the running motion properly, noting that different settings might yield different results.

At this point, we use some trial and errors to find a way to control actuators that will successfully make the half-cheetah run without using any neural network yet. Indeed, we found that the following works: (1) apply actuator with strengths $[0,0,0,-2s,0,0]$ for 25 time steps, (2) followed by $[-s,0,0,0,0,0]$ for the next 25 time steps (3) repeat step (1) and (2) cyclically. The rationale is simple: step (1) is used to swing the front thigh forward and step (2) the back thigh. With this simple test movements, the agent is able to run forward. We will refer to this as the basis of the neural network we use in SRD design.

Stage 1. In this stage, our goal is only to observe the position coordinates of half cheetah. Different strengths of actuators are applied to the half cheetah and then it is allowed to stabilize. A short video will be available for readers to verify half cheetah's pose visually. The final poses are shown in fig. 13. To proceed with the model design, execute the python command to run stage 1: `python mujoco_entry.py --mode srd-model-design --model half-cheetah --stage 1`. We will then save these positions in *init.params*, which we will use later.

In fig. 14(A), numbers 0 to 6 denote the 7 parts of half cheetah as defined in the xml format (known as the MJCF model

in the official MuJoCo documentation). For example, 0 corresponds to its torso, 1 to back thigh etc. The x and z position coordinates of these body parts relative to the torso's coordinates will be used as the input to the neural network that controls our SRD half-cheetah model, as in fig. 6(A). By observing the figure and the aforementioned short video, we verify that the agent is indeed initiated from a short distance above the ground, after which it will fall to the ground and stabilize on its front and rear legs.

Stage 2. In this stage, we consider how to convert the input (x and z coordinates) to a set of meaningful neuron activations. We start by considering neurons that respond to the stable standing pose from the previous stage. To achieve this, we use StablePoseNeuron, a custom pytorch module with parameter p and a forward propagation method that takes in input x and outputs $\sigma_{sa}((x-p)^2)$. In fig. 6(A), xS and zS are both StablePoseNeuron, while $xS_{inv} = 1 - xS$ and $zS_{inv} = 1 - zS$. When the agent stays in a stable pose, xS, zS will activate strongly while their corresponding inverses xS_{inv}, zS_{inv} are not activated, and vice versa. This is shown in fig. 14(B). In the scenario, the cheetah drops from a short height above the ground and stays in the equilibrium position until time step 250. From this point onward, the actuator of the agent's front thigh is activated, causing a forward swing of the front limb. This motion leads the agent away from its initial stable pose, and, as expected, the xS, zS neurons' signals drop off (and their inverses activate strongly). The goal of our stage 2 design has been achieved. Note: the command is the same as before, but with argument `--stage 2`.

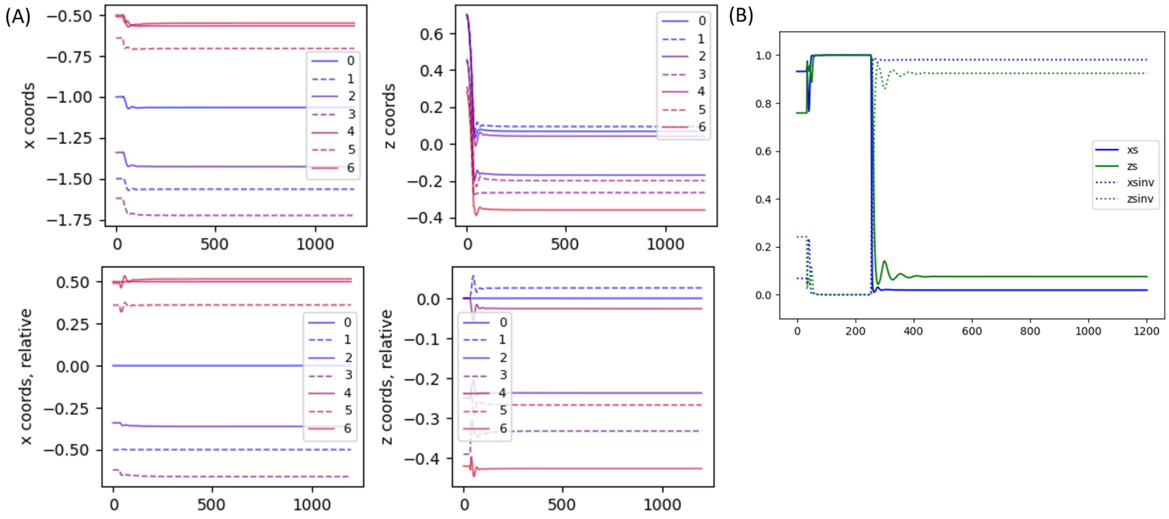

Figure 14. (A) x and z position coordinates of the agent, half cheetah, in stage 1. Numbers 0 to 6 denote the 7 parts of half cheetah (B) The strength of activation of stable pose neurons xS, zS over time, and likewise of their inverses.

Stage 3. In this stage, we connect the stable pose neurons and their inverses to the actuators. At this point, we have a neural network structure similar to the blue dotted box in fig. 6(A), except without the BS neuron. Recall in stage 0 we swing back and front thigh alternately. Our aim here is to approximately replicate the set up, and then upgrade it with a neural network. Thus HalfCheetahSRD is born, in which the parameters of other actuators can be optimized in the SRD way as we have done before. More specifically, a fully connected layer connects the stable pose neurons to the actuators bt, bs, bf, ft, fs, ff as shown in fig. 6(A). The simulation is then run similar to the previous stage. We implement the *momentum* function that ensures that the actuators apply their forces for 25 time steps before the next set of actuator's values are computed from the new, updated pose.

The results are shown in fig. 15(A). In essence, the plot of x coordinates shows that the agent moves forward successfully. The z coordinates drop from a height, as expected, and then oscillates regularly, indicating that the agent's body parts move in a regular cycle at a given level above the ground i.e. the cheetah does not trip or fly away etc.

Note: the command is the same as before, but with argument `--stage 3`.

Stage 4. Stage 4 is similar to 3, except with the introduction of BS neuron with the modification of `propagate_first_layer` function. This neuron creates an additional variable used to vary the movement pose of the agent. More specifically, it allows the agent to vary how much its hind thigh swings throughout the motion. One such result with $backswing = 5$ is shown in fig. 15(B).

The main experiment. The SRD is not yet complete without the PFC for SRD optimization. In this experiment, we keep the PFC simple, as shown in the green dotted box of fig. 6(A). As before, we want PFC to decide the correctness of the agent's action. The iN neuron responds to the inhibitor, where the inhibitor takes the value of either 0 or 2. When $inhibitor = 0$,

the agent will default to forward movement. But when $inhibitor = 2$, the agent is expected to stop moving. Without SRD optimization, the movements are plotted in fig. 6(C,D) bottom.

The PFC is designed such that the true T neuron activates more strongly when the actuator neuron Ac is activated while the iN neuron is not active. The false F neuron is approximately its reverse, penalizing motion when the inhibitor is active, i.e. when the controller wants the model to stop moving but the agent tries to move. The cross-entropy loss can be computed as before $CEL(z, argmax(z))$, and the results are what we have discussed in the main text.

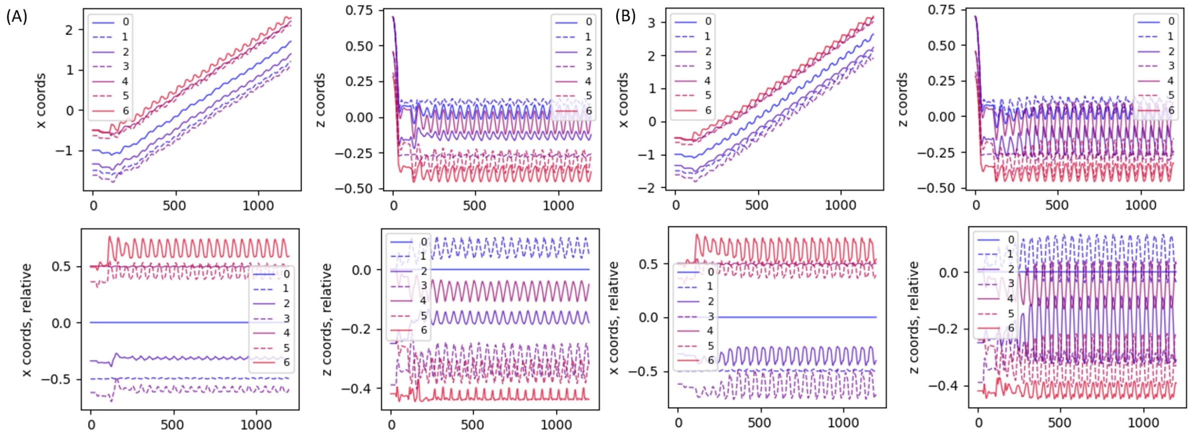

Figure 15. (A) x and z position coordinates of the agent in stage 3. (B) x and z position coordinates of the agent in stage 4.
